# The lncRNA ‘UCA1’ modulates the response to chemotherapy of ovarian cancer through direct binding to miR‐27a‐5p and control of UBE2N levels

**DOI:** 10.1002/1878-0261.13045

**Published:** 2021-07-13

**Authors:** Anaïs Wambecke, Mohammad Ahmad, Pierre‐Marie Morice, Bernard Lambert, Louis‐Bastien Weiswald, Mégane Vernon, Nicolas Vigneron, Edwige Abeilard, Emilie Brotin, Martin Figeac, Pascal Gauduchon, Laurent Poulain, Christophe Denoyelle, Matthieu Meryet‐Figuiere

**Affiliations:** ^1^ UNICAEN Inserm U1086 ANTICIPE (Interdisciplinary Research Unit for Cancer Prevention and Treatment) Normandie Univ Caen France; ^2^ Cancer Centre François Baclesse UNICANCER Caen France; ^3^ CNRS Normandy Regional Delegation Caen France; ^4^ ImpedanCELL Core Facility Federative Structure 4206 ICORE UNICAEN Caen France; ^5^ Functional and structural genomics platform Institute for Cancer Research Lille Univ France

**Keywords:** ceRNA, chemoresistance, miR‐27a‐5p, ovarian cancer, patient‐derived organoid, lncRNA UCA1

## Abstract

Ovarian cancer (OC) is the leading cause of death in patients with gynecologic cancers. Due to late diagnosis and resistance to chemotherapy, the 5‐year survival rate in patients with OC is below 40%. We observed that UCA1, a lncRNA previously reported to play an oncogenic role in several malignancies, is overexpressed in the chemoresistant OC cell line OAW42‐R compared to their chemotherapy‐sensitive counterpart OAW42. Additionally, UCA1 overexpression was related to poor prognosis in two independent patient cohorts. Currently, the molecular mechanisms through which UCA1 acts in OC are poorly understood. We demonstrated that downregulation of the short isoform of UCA1 sensitized OC cells to cisplatin and that UCA1 acted as competing endogenous RNA to miR‐27a‐5p. Upon UCA1 downregulation, miR‐27a‐5p downregulated its direct target UBE2N leading to the upregulation of BIM, a proapoptotic protein of the Bcl2 family. The upregulation of BIM is the event responsible for the sensitization of OC cells to cisplatin. In order to model response to therapy in patients with OC, we used several patient‐derived organoid cultures, a model faithfully mimicking patient’s response to therapy. Inhibition of UBE2N sensitized patient‐derived organoids to platinum salts. In conclusion, response to treatment in patients with OC is regulated by the UCA1/miR‐27a‐5p/UBE2N axis, where UBE2N inhibition could potentially represent a novel therapeutic strategy to counter chemoresistance in OC.

## Graphical abstract

UCA1 cytoplasmic short isoform is acting as a competing endogenous RNA for miR‐27a‐5p. Upon UCA1 down‐regulation, miR‐27a‐5p is no longer prevented to bind and downregulate its direct target UBE2N. UBE2N inhibition triggers *BIM* upregulation, which sensitizes cells and patient derived organoids to the cytotoxic action of platinum drugs.

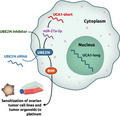

**Abbreviations**
ATCC: American Type Culture Collection
BRCA1/2: breast cancer type 1/2 susceptibility protein
CeRNA: competing endogenous RNA
CUDR: cancer upregulated drug resistant transcript
DEGs: differentially expressed genes
ECACC: European Collection of Authenticated Cell Cultures
EOC: epithelial ovarian cancers
GO‐BP: gene ontology‐biological processes
HR: homologous recombination
OC: ovarian cancer
PARP: poly (ADP-ribose) polymerase
PARPi: poly (ADP-ribose) polymerase inhibitor
PDL-1: programmed death-ligand 1
PFS: platinum free survival
UBE2N: ubiquitin conjugating enzyme E2N
UBE2Ni: ubiquitin conjugating enzyme E2N inhibitor
UCA1: urothelial cancer associated 1

## 1 Introduction

Ovarian cancers are the leading cause of death from gynecologic malignancies. They represent the 7^th^ most frequent cancer in women worldwide and are responsible for around 240 000 new cases and 152 000 deaths annually [1]. The standard treatment for EOC (epithelial ovarian cancers) consists of cytoreductive surgery and platinum salts/taxane chemotherapy [2]. Its asymptomatic nature during the early stages generally leads to a late diagnosis. Despite good initial response rate, the overall 5‐year survival rate is below 40% for the advanced FIGO stages (III/IV) because of recurrence and resistance to conventional therapy [3]. Recently, several malignancies have benefited from the introduction of innovative and targeted therapies, but ovarian cancer has lagged behind until recently. The newly introduced PARP inhibitors are highly promising, but to only a subset of *BRCA1/2* mutated and/or homologous DNA repair‐deficient patients, who still show sensitivity to platinum salts chemotherapy [4]. Many efforts are set up to counter the chemoresistance of ovarian cancer, which remains the major hurdle for a significant improvement of outcome.

The identification of the roles and functions of noncoding RNAs has shed new light on the regulation of gene expression. At first, the discovery of microRNAs (miRNAs) and the subsequent characterization of their functions evidenced their crucial roles in all of the main biological processes and in many diseases including cancer, where they can act as oncogenes or tumor suppressors [5, 6]. More recently, a large number of long noncoding RNAs (lncRNAs) that have been described with 5946 lncRNA genes were identified in the LNCipedia database [7, 8]. LncRNAs lack a significant ORF and are longer than 200 nucleotides and are involved in most biological processes, where they regulate gene expression at epigenetic, transcriptional, and post‐transcriptional levels [9]. Furthermore, lncRNAs have the ability to prevent miRNAs from performing their regulatory functions on their target mRNAs by fixing them, acting as sponges of miRNAs [10]. Deregulation of their expression is associated with tumorigenesis and prognosis in many cancers, including ovarian carcinoma [11], where they can act as oncogenes or tumor suppressors.

Human urothelial carcinoma‐associated 1 (UCA1) has 3 exons, and two main isoforms have been described of 1.4 and 2.2 kb, differing only by the length of the 3^rd^ exon [12]. Studies exploring UCA1 functions have been expanding rapidly in the recent years, although most of them do not attribute UCA1 functions to a specific isoform. UCA1 is an oncofetal gene with oncogenic functions reported in many cancers [13]; for instance, an increase in UCA1 expression promotes proliferation and metastases formation in bladder cancer [14] and is a factor of poor prognosis in colorectal cancer and glioma [15, 16]. UCA1 has been involved in resistance to several chemotherapeutic drugs in different tumor types [13], including in ovarian cancer [17].

Out of the several studies about UCA1 functions, a majority does not propose mechanistic explanations for the observed effects. It has however been demonstrated that UCA1 is able to interact with BRG1, a member of the SWI/SNF chromatin‐modifying epigenetic complex [18], or to act as a competing endogenous RNA for several miRNAs [19, 20]. In ovarian cancer, UCA1 has been shown to directly interact with miR‐129 and thus control the expression of ABCB1 protein, leading to the resistance to paclitaxel [21].

In this study, UCA1 was identified as promoting the chemoresistance of ovarian cancers. We showed that UCA1 binds directly to miR‐27a‐5p. Upon UCA1 downregulation, miR‐27a‐5p is free to downregulate UBE2N expression through direct targeting, resulting in the upregulation of proapoptotic BCL2 family protein BIM. BIM upregulation efficiently sensitizes ovarian cancer cell lines to the action of cisplatin. Additionally, we showed that UBE2N inhibition in patient‐derived organoid cultures also triggers sensitization to cisplatin, enlightening a potentially promising innovative therapeutic strategy.

## 2 Material and methods

### 2.1 Ovarian cancer cell lines

The cisplatin‐sensitive OAW42 serous ovarian cancer cell line was obtained from ECACC (Sigma‐Aldrich, St Quentin‐Fallavier, France). Its cisplatin‐resistant counterpart OAW42‐R was obtained as previously described [22]. Briefly, OAW42 cells were exposed to increased concentrations of cisplatin and were allowed to recover in‐between treatments. Over time, acquired resistance to cisplatin was established and the resultant cell line was termed ‘OAW42‐R’. OAW42 and OAW42‐R cells were grown in DMEM supplemented with 4500 mg·L^−1^ glucose, 2 mm Glutamax™, 1 mm sodium pyruvate, 10% fetal bovine serum, 33 mm sodium bicarbonate (Gibco BRL, Lyon, France), and 20 UI·L^−1^ recombinant human insulin (NovoRapid (insulin aspartate); Novo Nordisk, Puteaux, France). OVCAR3 ovarian cancer cell line was obtained from ATCC (LGS Standards) and was grown in RPMI1640 medium supplemented with 2 mm Glutamax™, 10% fetal bovine serum, 20 mm HEPES, and 33 mm sodium bicarbonate. All cell lines were maintained at 37 °C in a 5% CO2 humidified atmosphere.

### 2.2 Organoids

#### 2.2.1 Patient samples

Fresh ascitic samples were collected from patients treated at Centre Francois Baclesse (Unicancer center, Normandy) for High‐Grade Serous Ovarian Cancer between September 2018 and January 2019. All three patients presented an incomplete response to platinum‐based chemotherapy. Informed consent forms were signed by all patients (n = 3) and were obtained by Biological Resource Centre (CRB OvaRessources NF S96‐900), in accordance with ethical committee and European law.

#### 2.2.2 Ascites processing and organoid culture

Ascites were spun at 430 ***g*** to create cell pellets. Pellets were resuspended in ‘collecting medium’ [RPMI1640 medium supplemented with 2 mm Glutamax™, 25 mm HEPES (Gibco), 33 mm sodium bicarbonate (Gibco), 100 UI·mL^−1^ of penicillin (Gibco), 100 µg·mL^−1^ of streptomycin (Gibco), and 1% bovine serum albumin (Sigma‐Aldrich)]. Tumor spheroids of 50–300 μm in diameter were selected using cell strainers (Endecotts, Garches, France) and enzymatically dissociated into single cells/small cell clusters using TrypLE Express (Gibco) for up to 10 min at 37 °C. The cell pellets were washed once with collecting medium and resuspended in a small volume of organoid culture medium and mixed with a 1 : 1 volume of growth factor‐reduced Matrigel (Corning). Drops of 50 µL of Matrigel/cell suspension (one drop and 10 000 cells per well) were distributed into a prewarmed 24‐well plate (Eppendorf, Montesson, France). Once the Matrigel was solidified, 500 μL of organoid culture medium was added to each well. The medium was changed twice a week, and tumor organoids were passaged every 7–14 days by dissociation with TrypLE Express for up to 10 min at 37 °C. Single cells and small cell clusters were replated according to the procedure described above. Cryovials were prepared at regular intervals by dissociating and resuspending organoids in Recovery Cell Culture Freezing Medium (Gibco) prior to be biobanked in liquid nitrogen.

#### 2.2.3 Organoid media

Patient‐derived organoids were cultured in advanced DMEM (Gibco) supplemented with 100 UI·mL^−1^ of penicillin, 100 µg·mL^−1^ of streptomycin, 1% GlutaMAX (Gibco), 1X B27 (Gibco), 1.25 mm NAC (Sigma‐Aldrich), 50 ng·mL^−1^ EGF (PeproTech, Neuilly sur Seine, France), 20 ng·mL^−1^ FGF‐10 (PeproTech), 1 ng·mL^−1^ FGF‐2 (PeproTech), 500 nm A‐83‐01 (PeproTech), 10 µm Y‐27632 (Interchim, Montluçon, France), 1 µm SB202190 (PeproTech), 10 mm Nicotinamide (Sigma‐Aldrich), 1 µm PGE2 (PeproTech), 100 µg·mL^−1^ Primocin (InvivoGen, Toulouse, France), 50% Wnt3a, RSPO3, Noggin‐conditioned media (L‐WRN, ATCC), and 10% RSPO1‐conditioned media (Cultrex HA‐R‐Spondin‐1‐Fc 293T, Amsbio, Abingdon, UK).

#### 2.2.4 UBE2N inhibition assays

Organoid culture medium lacking N‐Acetylcysteine, Y‐27632, and primocin was mixed with a 1 : 1 volume of growth factor‐reduced Matrigel, and 45 µL of the solution was dispensed into white, clear‐bottom 96‐well plates (Greiner, Les Ulis, France). Tumor organoids were collected before being resuspended in 2% Matrigel/organoid culture medium lacking N‐Acetylcysteine, Y‐27632, and primocin and plating in 100 µL volume. UBE2N inhibitor (Santa Cruz, Clinisciences, Nanterre, France, NSC697923) was added in triplicate wells 1 h after plating organoids. Three days postseeding, media was replaced by a final volume of 200 µL supplemented with UBE2Ni alone (or DMSO, Sigma), or with a combination of UBE2Ni and a range of carboplatin (Accord Healthcare, Lille, France) concentrations (or with DMSO, Sigma) diluted in adapted organoid media. Eight days later, ATP levels were quantified using CellTiter‐Glo 3D cell viability assay (Promega, Charbonnières les Bains, France) according to the manufacturer’s instruction, and luminescence was measured using Centro XS3 LB 960 (Berthold Technologies, Thoiry, France) with miko win 2000 software. All viability results were normalized to DMSO. Real‐time monitoring of organoids (growth, size, and morphology) was performed by the IncuCyte S3 (Sartorius, Aubagne, France).

### 2.3 Transfection

Exponentially growing cells were seeded at 150 000 cells (OAW42 and OAW42‐R cells) or 350 000 cells (OVCAR3) per 25 cm^2^ flask. Twenty‐four hours after seeding, siRNA or miRNA were diluted in OptiMEM^®^ (Gibco) and transfection complexes were formed with INTERFERin™ (Polyplus‐Transfection, Illkirch, France). We used a negative siRNA control (Lincode Non‐targeting Pool; D‐001320‐10‐05 Dharmacon) noted sictrl, and siRNAs targeting UCA1 were labeled as follows: ‘siUCA1’ or ‘siUCA1‐total’ constituted of both siUCA1(2) and siUCA1(4) targeting both the short and long isoforms and siUCA1‐long constituted of siUCA1(3) targeting specifically the long isoform. siRNA targeting UBE2N was noted siUBE2N. These siRNAs were purchased from Dharmacon. siRNA targeting BIM noted siBIM was purchased from Eurogentec (Seraing, Belgium). Human miRNA hsa‐miR‐27a‐5p‐Mimic (C‐301028‐01‐0005) and mimic negative controls cel‐miR‐67 (CN‐001000‐01‐05) were purchased from Dharmacon. Sequences of siRNAs and miRNAs are presented in Table S1.

### 2.4 Drug treatments

OAW42 and OAW42‐R cells were reseeded at 250 000 cells per 25 cm^2^ flask 48 h after transfection. Next day, 72 h after transfection, they were treated with cisplatin and the sensitization was monitored 72 h later (6 days after transfection). OVCAR3 cells were treated 24 h after transfection, and the sensitization was monitored 72 h after transfection. Cisplatin was obtained from Mylan (Merck, France). For cisplatin treatment conditions, cells were exposed to serum‐free cisplatin supplemented medium for 2 h (or serum‐free medium for control conditions) and then were incubated back again in the complete growth medium during the indicated time. OAW42 was treated with 5 µg·mL^−1^ of cisplatin, OAW42‐R with 20 µg·mL^−1^ of cisplatin, and OVCAR3 with 2.5 µg·mL^−1^ of Cisplatin.

UBE2N inhibitor (noted UBE2Ni) was purchased from Santa Cruz (NSC697923; ref: SC‐391107), and stock solution was prepared in sterile dimethyl sulfoxide (DMSO; Sigma‐Aldrich) according to the manufacturer’s instructions, with a final concentration of 10 mm and stored at −20 °C. OAW42‐R were treated with continuous exposure of UBE2Ni‐supplemented growth medium (DMSO supplemented for control conditions), starting from the time of transfection onward.

### 2.5 Western blotting

Proteins were extracted as follows: Cells were washed with ice‐cold PBS 1X, lysed in lysis buffer (15 mm HEPES, 50 mm KCl, 10 mm NaCl, 1 mm MgCl2, 0.25% glycerol, 0.5% n‐Dodecyl‐β‐d‐maltopyranoside (DDM) (Affymetrix), 5 µm GDP (Sigma‐Aldrich), 1 µm microcystin (EnzoLifeSciences, Villeurbanne, France), 1 mm Na_3_VO_4_ (Sigma‐Aldrich), and Complete Protease Inhibitor Cocktail (Roche, Boulogne Billancourt, France)), and incubated on ice for 30 min. After centrifugation (7826 ***g***; 10 min) to remove cell debris, proteins were quantified using the Bradford assay (Bio‐Rad, Hercules, CA, USA). Fifteen micrograms of proteins were separated by SDS/PAGE (Bio‐Rad) on a 4–15% gradient polyacrylamide Mini‐PROTEAN^®^ TGX™ precast gel (Bio‐Rad) and transferred to PVDF membranes (Bio‐Rad). After blocking during 1 h at room temperature with 5% (v/v) nonfat dry milk in TBS with 0.05% (v/v) Tween 20 (T‐TBS), membranes were incubated overnight at 4 °C with the following primary antibodies: anti‐actin (MAB1501, Merck Millipore, Sigma Aldrich, St Quentin Fallavier, France), anti‐BIM (2819, Cell Signaling Technology, Ozyme, St Cyr l'Ecole, France), and anti‐UBC13 (UBE2N) (37‐1100; Thermo Fisher, Illkirch, France). Membranes were then incubated with the appropriate horseradish peroxidase‐conjugated anti‐rabbit (70745; Cell Signaling Technology) or anti‐mouse (NA931V; Amersham, Les Ulis, France) secondary antibodies. Signals were revealed using EleCtroLuminescence (ECL) Prime Western Blot detection reagent (GE Healthcare Life Sciences, Velizy Villacoublay, France) under ImageQuant^®^ Las4000Series imager (GE Healthcare Life Sciences) and then quantified by pixel densitometry using the imagej ^®^ software (NIH, free download at: https://imagej.nih.gov/ij/download.html). Western blots shown are from one experiment representative of at least three independent experiments.

### 2.6 Cell cycle and apoptosis analysis by flow cytometry

Cells were detached with trypsin, harvested and washed with 1X PBS (phosphate‐buffered saline), fixed in 70% ethanol, and stored at −20 °C. Before the analysis, fixed cells were centrifuged (1252 ***g***, 5 min) and incubated for 3 min at 37 °C in PBS. After centrifugation, cell pellets were dissociated and incubated with RNAse and propidium iodide using the DNA‐Prep Coulter Reagent kit (Beckman Coulter, Villepinte, France) and were analyzed with a Gallios flow cytometer (Beckman Coulter). A computerized gating was applied on the side and front diffusion to exclude small debris and on a pulse width and an integral red fluorescence peak to remove aggregates. The data were analyzed by the Kaluza^®^ acquisition software (Beckman Coulter).

### 2.7 RNA extraction and real‐time quantitative reverse transcription PCR (RT‐qPCR)

Total RNA was isolated from cells using a NucleoSpin RNA+ kit (Macherey‐Nagel, Hoerdt, France; 74098450) according to the manufacturer’s instructions. RNA was eluted from columns in nuclease‐free water, dosed, and quality‐controlled on a NanoDrop™ 2000 spectrophotometer (Thermo Scientific). Gene expressions were determined by RT‐qPCR; at first, 1 µg of total RNA extracted was reverse‐transcripted with Omniscript^®^ reverse transcriptase kit (Qiagen, Courtaboeuf, France), Buffer 10X, dNTPs, RTase (enzyme Omniscript Reverse Transcriptase 4 units/20 μL), random hexamers primers (Invitrogen; 48190.011), and RNase inhibitor (RNaseOUT; 100000840; Invitrogen). RNA in solution was incubated at 65 °C for 5 min, cooled rapidly, and incubated at 4 °C for 5 min. The reaction mix was then added and RT reaction took place at 37 °C for 60 min and was stopped at 93 °C for 5 min.

PCR amplification was performed in triplicate, 5 µL of MIX 2X (Light Cycler^®^480 SYBR^®^ Green I Master, Roche, 04707516001), 1.9 µL of water, 0.3 µL of each forward and reverse primers (Eurogentec), and 2.5 µL of cDNA for a 10 µL final reaction volume. PCR program was as follows: 95 °C 5 min, 40X (95 °C 15 s, 60 °C 30 s, 72 °C 30 s). Melting curves were established in the end. All PCR amplification reactions were carried on a Light Cycler^®^480 Real‐Time PCR instrument, and analysis was done with the Light Cycler^®^480 software release 1.5.1.62. Data are representative of three independent experiments performed in triplicate, and relative change expressions were calculated by the 2^‐ΔΔCq^ with SDHA as a control gene. Primer sequences are represented in Table S2.

### 2.8 Pull‐down assay

Three 25 cm^2^ flasks of OAW42‐R cells (200 000 cells) were transfected with 3`‐Biotin‐labeled miRNAs (Biotin‐Cel‐miR‐67‐3p (Control) or Biotin‐miR‐27a‐5p) at a final concentration of 60 nm. T1 streptavidin beads (Invitrogen) were washed with 1X washing buffer (20 mm Tris‐HCl, pH = 7.5, 5 mm MgCl2, 100 mm KCl, 0.3% Igepal CA‐630) and incubated with rotation for 2 h at 4 °C using 1x blocking buffer (1X washing buffer, 50 mg·mL^−1^ BSA, 10 mg·mL^−1^ yeast t‐RNA) before pull‐down. Twenty‐four hours after transfection, cells were harvested and lysed (lysis buffer: 1X washing buffer, 50X protease inhibitor, 100 U RNAse inhibitor). Cell lysate was rotated with blocked beads for 4 h at 4 °C, and then, beads were washed twice in washing buffer and captured RNA was extracted using TRIzol reagent. RT‐qPCR was performed for RNA samples and pull‐down fold enrichment was calculated with respect to SDHA as a control gene. Fold‐enrichment calculation was as follows: (Biotin‐miR‐27a‐5p pull‐down/ Biotin‐miR‐27a‐5p input) / (Biotin‐Cel‐miR‐67 pull‐down/Biotin‐Cel‐miR‐67 input). This pull‐down protocol for biotinylated miRNA‐bound RNAs was first published by Lal *et al*. [23].

### 2.9 Microarray

#### 2.9.1 For OAW42‐R and OAW42 untreated cell lines

RNA concentration was assessed using the Qubit reagent assay RNA quantification kit (Thermo Fisher Scientific). 100 ng RNA per sample was retro‐transcribed to biotin‐labeled single‐strand cDNA (ss‐cDNA) with the Affymetrix Genechip WT Plus. 5.5 µg ss‐cDNA were hybridized to Affymetrix arrays (Genechip HTA human array), and data obtained were normalized by RMA process for the whole set of samples (Affymetrix Expression Console). Probe‐set annotation, quantitative expressions of all the transcripts, and comparisons between the different groups of samples were analyzed using the affymetrix software TAC.v3 (Thermofisher).

#### 2.9.2 For UCA1 downregulation experiment in OAW42‐R cells

Total RNA from sictrl‐ or siUCA1‐transfected cells (72 h after transfection) from four independent experiments was isolated from ovarian cancer cell lines using a NucleoSpin RNA+ kit. RNA was resuspended in nuclease‐free water, dosed, and quality‐controlled on a NanoDrop™ 2000 spectrophotometer. RIN value was determined on a bioanalyzer 2000 with the RNA Pico kit. One‐color whole Human 60‐mer oligonucleotides (8x60k) microarrays (Agilent Technologies) were used to analyze gene expression. cRNA labeling, hybridization, and detection were carried out according to supplier’s instructions (Agilent Technologies). For each microarray, cyanine 3‐labeled cRNA was synthesized with the low‐input QuickAmp labeling kit from 50 ng of total RNA. RNA spike‐in was added to all tubes and used as positive controls of labeling and amplification steps. The labeled cRNAs were purified and 600 ng of each cRNA was then hybridized and washed following manufacturer’s instructions. Microarrays were scanned on an Agilent G2505C scanner and data extracted using Agilent Feature Extraction Software. *P*‐values for differential expression were processed with moderated *t*‐test using Limma R Package and corrected with Benjamini–Hochberg.

### 2.10 Bioinformatics

Pathway analysis for DEGs after UCA1 downregulation was performed at DAVID [24] website (V6.7) and interrogated Gene Ontology‐Biological Processes database. Enrichment of miRNAs seed regions in the 3’UTR of DEGs was analyzed at GSEA [25] website (C3 section: motif gene sets, MIR: microRNA targets). The list of miRNAs whose seed regions were enriched was overlapped with the miRNAs predicted to bind to UCA1 according to RegRNA 2.0, MiRTarBase (microRNA‐target interactions database), Starbase, and RNA22 v2 websites and whose binding was reported in the literature referenced in PubMed.

The prognostic value of UCA1 expression levels in tumors was assessed using the kmplot tool (https://kmplot.com/). For indicated cohorts, the parameters were as follows: ovarian cancer serous subtype only, treatment contains platinum, best cutoff selection, and UCA1 probeset: 227919_at.

### 2.11 Cell confluency measurements

Pictures of cell layer in culture flasks were analyzed with ImageJ software (ImageJ bundled with 64‐bit Java 1.8.0_172, freely available at https://imagej.nih.gov/ij/download.html). Total area of the picture and cell surface area were measured. Confluency was calculated as the ratio of [cell surface area]/[total area] and expressed percent confluency, ± SD from 3 experiments.

### 2.12 Statistical analysis

Statistical significance was calculated with Student’s two‐tailed *t*‐test, with *P*‐values < 0.05 noted as significant. *P* < 0.05 was noted as ‘*’, and *P* < 0.01 was noted as ‘**’. Calculations were made on Excel software. For bioinformatic analyses, statistical significance calculations and thresholds were defined following the guidelines of the online tools we used, that is, FDR adjusted *P*‐values below 0.05 were considered as significant (see also Bioinformatics section in the material and methods).

### 2.13 Ethics approval and consent to participate

Informed consent forms were signed by all patients from whom tumor samples were taken to generate organoids and were obtained by Biological Resource Centre (CRB OvaRessources NF S96‐900), in accordance with ethical committee, European law. The study methodologies conformed to the standards set by the Declaration of Helsinki.

## 3 RESULTS

### 3.1 LncRNA UCA1 high expression is a factor of bad prognosis and its downregulation sensitizes chemoresistant ovarian cancer cells to cisplatin

**Fig. 1:**
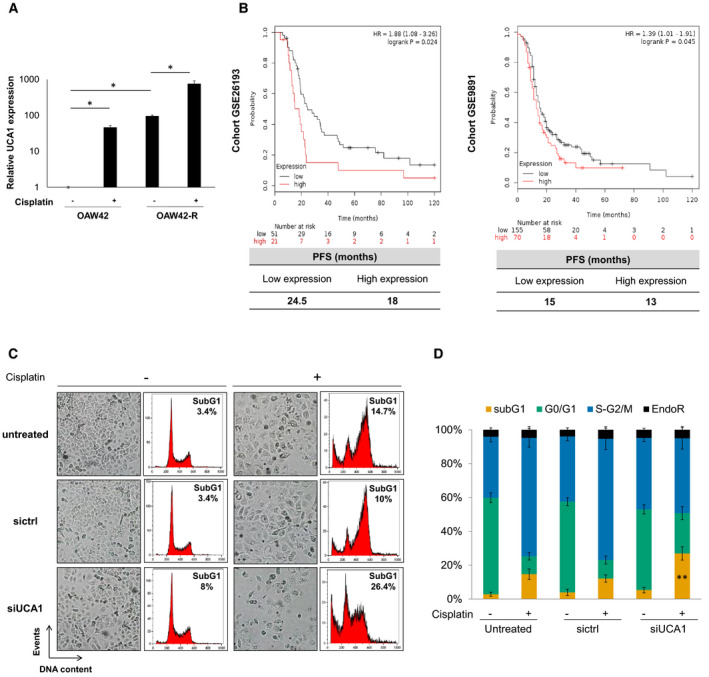
High UCA1 is a poor prognosis factor and its inhibition sensitizes OAW42‐R cells to cisplatin. (A) UCA1 expression levels (RT‐qPCR) for OAW42 and OAW42‐R cells, with or without cisplatin treatment (5 and 20 µg·mL^−1^ for OAW42 and OAW42‐R, respectively), relative to untreated OAW42, normalized with SDHA. Results are expressed as mean values ± SEM of 3 experiments. (B) Kaplan–Meier curves generated with KM plot [57] (Kaplan–Meier plotter) software, showing the progression‐free survival (PFS), for two cohorts of patients with serous ovarian cancer treated with platinum salts (GSE26193, *n* = 72; GSE9891, *n* = 225). (C) Picture from cell layer and DNA content profiles. Representative pictures and profiles from 3 experiments are shown. (D) DNA content histograms expressed as mean ± SEM of 3 experiments. *P < 0.05; **P < 0.01

We compared the transcriptomic profiles between an ovarian cancer cell line sensitive to cisplatin (OAW42) and its resistant counterpart (OAW42‐R). Out of the 1717 differentially expressed genes (DEGs) were 16 lncRNAs. UCA1 was the most upregulated lncRNA in the resistant cell line, with a fold change of 40. This was validated by RT‐qPCR, with UCA1 expression around 100‐fold higher in OAW42‐R cells compared to OAW42 cells (Fig. 1A). In addition, UCA1 expression was increased following cisplatin treatment in both cell lines (Fig. 1A).

We explored UCA1 expression levels in ovarian cancer tissues in publicly available data sets from two independent cohorts. For GSE26193 cohort, low‐expression group for UCA1 presents a longer median progression‐free survival (PFS) of 24.5 months versus 18 months for high‐expression group (*P* = 0.024). For GSE9891 cohort, low UCA1 expression group also presents a longer median PFS of 15 versus 13 months (*P* = 0.045) (Fig. 1B).

We next studied the effects of UCA1 inhibition on the response to cisplatin in OAW42‐R cell line. A cocktail of two siRNAs targeting UCA1, termed ‘siUCA1’ decreased UCA1 levels by 96% (Fig. S1A). UCA1 inhibition slightly decreased the number of cells, as seen by a less confluent cell layer, compared to untreated and control siRNA (sictrl) conditions (Fig. 1C). After cisplatin treatment, OAW42‐R cells in control conditions had undergone a phenotypic change characterized by cell swelling and were mostly blocked at the S‐G2/M phase of the cell cycle. A moderate increase in the percentage of sub‐G1 events in flow cytometry was also evidenced for untreated (15%) and sictrl (10%) conditions. In contrast, upon UCA1 inhibition, cisplatin largely decreased cell layer and increased cell death as shown by 27% of sub‐G1 events compared to 15% or 10% in untreated and sictrl conditions respectively (Fig. 1C,D).

In two other ovarian cancer cell lines, OAW42 and OVCAR3, both sensitive to cisplatin treatment, UCA1 expression levels were around 100‐ and 200‐fold lower respectively than in OAW42‐R (Fig. 1B).

In both cell lines, upon cisplatin treatment, UCA1 inhibition decreased the confluency of cell layer and increased cell death with a higher percentage of sub‐G1 events compared to untreated and sictrl conditions. For OAW42 cells, siUCA1 led to 43.9% sub‐G1 events versus 27% and 22.9% for untreated and sictrl conditions upon cisplatin treatment (Fig. 1C,D). For OVCAR3 cells, siUCA1 leads to 31.5% sub‐G1 events versus 20.9% and 19.3% in untreated and sictrl conditions (Fig. 1E,F).

### 3.2 UCA1 inhibition affects cell death‐related pathways

**Fig. 2:**
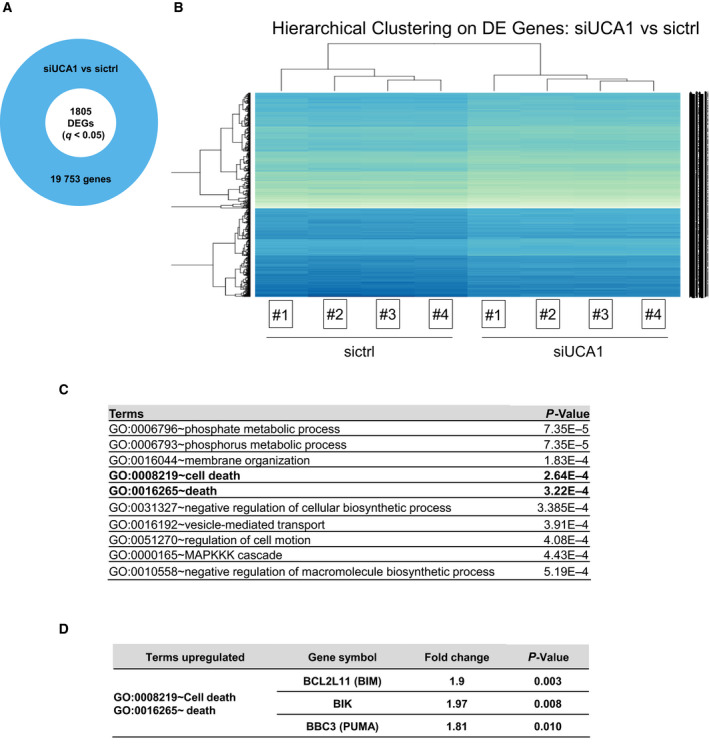
UCA1 inhibition influences cell death‐related pathways. (A) Out of 19 753 genes whose expression was quantified on microarrays, 1805 were differentially expressed (Benjamini–Hochberg corrected *P*‐value < 0.05) after siUCA1 transfection in OAW42‐R cells. (B) Hierarchical clustering of quadruplicate runs on microarray. (C) Top 10 enriched GO‐BP terms in the 1805 DEGs. (D) Fold changes and *P*‐values of upregulated transcripts of BH3‐only genes BIM, BIK, and PUMA

To determine how UCA1 acts on the chemoresistance of ovarian cancer cells, we compared the transcriptomic profiles of OAW42‐R cells 3 days after transfection with sictrl or siUCA1 using microarrays. We could analyze the expression of 19 753 genes, of which 1805 were significantly (*q* < 0.05) DEGs (Fig. 2A,B, Appendix S1). Among the most enriched Biological Processes in our DEGs according to Gene Ontology‐Biological Processes (GO‐BP) tool from DAVID [24] were some related to cell death (GO terms ‘cell death’ and ‘death’) (Fig. 2C).

Cell fate relative to death or survival is regulated through many pathways, leading to the control of expression and/or stability of members of the BCL2 family. When the balance of expression between pro‐ and antiapoptotic members of this family of proteins tips in favor of proapoptotic members, this leads to cell death. Among the genes annotated in the ‘death’ and ‘cell death’ terms were 3 proapoptotic members, upregulated upon UCA1 downregulation: BIM, BIK, and PUMA with fold changes (FC) of 1.9 (*q* = 0.003), 1.97 (*q* = 0.008), and 1.81 (*q* = 0.01), respectively (Fig. 2D and Appendix S1). We chose to investigate these based on their significant upregulation.

### 3.3 Cisplatin sensitization through UCA1 downregulation relies on BIM upregulation

**Fig. 3:**
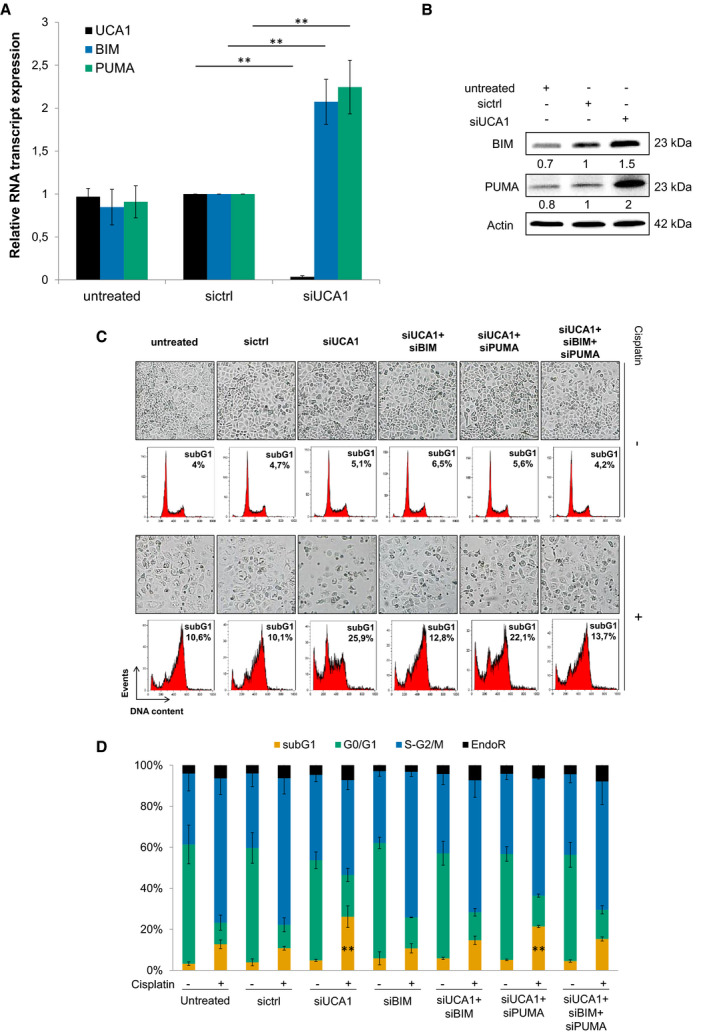
Cisplatin sensitization through UCA1 downregulation relies on BIM upregulation. (A) UCA1 BIM and PUMA transcript expression (RT‐qPCR) after indicated siRNA transfection in OAW42‐R cells, relative to sictrl, normalized with SDHA. Data are expressed as mean ± SEM of three independent experiments. (B) BIM and PUMA protein levels (western blot), quantified relative to actin loading control, representative or mean ± SEM of 3 experiments, after indicated siRNA transfection in OAW42‐R cells. (C) Picture from cell layer and DNA content profiles. Representative pictures and profiles are shown. (D) DNA content histograms expressed as mean ± SEM of 3 experiments. *P < 0.05; **P < 0.01

We validated that BIM and PUMA transcript and protein expression levels are increased 72 h after inhibition of UCA1 compared with control conditions (Fig. 3A,B), whereas BIK low expression levels prevented detection of its RNA and protein levels. We then investigated the possible involvement of BIM and PUMA overexpression in the sensitization of OAW42‐R cells to cisplatin caused by UCA1 inhibition. The transfection of siRNAs targeting BIM or PUMA (Fig. S2A) did not influence OAW42‐R cell response to cisplatin (Fig. 3C,D, and Fig. S2B,C). When siPUMA was used in combination with siUCA1, it did moderately reduce the sensitization to cisplatin induced by siUCA1, as observed on the cell layer and sub‐G1 events (Fig. 3C,D). However, when siBIM was used in combination with siUCA1, it abrogated the sensitization to cisplatin otherwise observed upon UCA1 downregulation alone. Indeed, cell layer confluency and proportion of sub‐G1 events show similar levels between control conditions and siUCA1 associated with siBIM (Fig. 3C,D, and Fig. S2A,B).

### 3.4 UCA1 short isoform inhibition, and not that of UCA1 long isoform, is responsible for sensitization to cisplatin

**Fig. 4:**
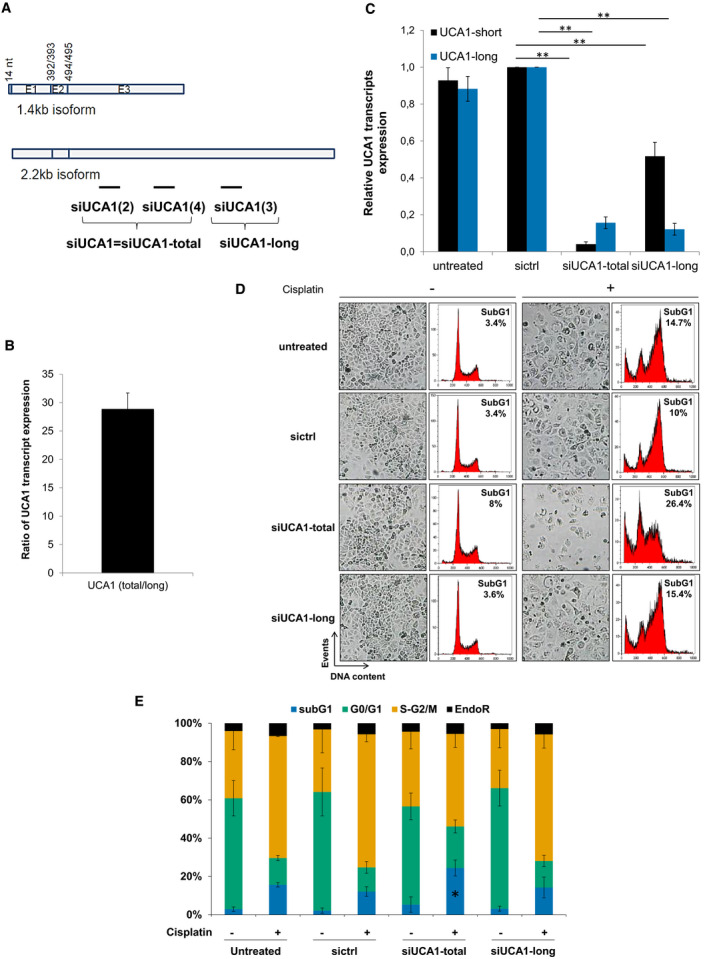
UCA1 short isoform inhibition is responsible for cisplatin sensitization. (A) Schematic representation of the short and long isoforms of UCA1, respectively, 1.4 and 2.2 kb, with the location of siRNAs used in this study. We used a siRNA pool composed of 2 siRNAs annotated ‘siUCA1(2)’ and ‘siUCA1(4), both targeting UCA1 short and long isoforms and named ‘siUCA1’, or ‘siUCA1‐total’. We used also a siRNA targeting only the long isoform, annotated ‘siUCA1(3)’ and named ‘siUCA1‐long’. (B) Ratio of UCA1 total and UCA1 long transcripts expression (RT‐qPCR) normalized to SDHA in OAW42‐R cells. Data are expressed as mean ± SEM of 3 independent experiments. (C) Relative UCA1 total and UCA1 long transcripts expressions (RT‐qPCR), normalized to SDHA, relative to sictrl in OAW42‐R cells, 72 h after transfection with indicated siRNAs. Data are expressed as mean ± SEM of 3 experiments. (D) Pictures from cell layer and DNA content. Representative pictures and profiles are shown. Data are representative of 3 experiments. (E) DNA content histograms expressed as mean ± SEM of 3 experiments. *P < 0.05; **P < 0.01

At least two isoforms of UCA1 have been described: the shorter one is 1.4 kb in length and was termed ‘short isoform’, and the longer one is 2.2 kb and was termed ‘long isoform’ in this study. These two isoforms only differ by a 5’ extension of 14 nt in the short isoform, and the size of the 3^rd^ and last exon [26] (Fig. 4A). In order to discriminate them, we wanted to use siRNAs and primers able to target each isoform independently (Fig. 4A). We failed to target the short isoform only by designing siRNAs and primers encompassing the unique 5’ extension of 14nt. By comparing the ‘total’ versus ‘long only’ expression levels, we could demonstrate in OAW42‐R cells that the short isoform of UCA1 is predominantly expressed compared to the long isoform, by approximately 29‐fold (Fig. 4B). Since the majority of the signal amplified by ‘UCA1 total’ primers represent the short isoform, it provides a suitable proxy short isoform quantification.

Our siRNA cocktail targets both UCA1 isoforms with 96% and 84% inhibition for short and long isoform, respectively (Fig. 4C). We designed a siRNA able to target the long isoform only, with 88% downregulation efficiency, which only moderately downregulated the short isoform by 48% (Fig. 4C). We thus determined which isoform of UCA1 was involved in cisplatin sensitization in OAW42‐R cells. UCA1 long isoform inhibition before cisplatin treatment did not induce any change in the cell layer or proportion of sub‐G1 events (14%) compared to control conditions (16% and 12% for untreated and sictrl, respectively), while the inhibition of both isoforms did induce sensitization, as usually (Fig. 4D,E). We can conclude that the inhibition of the short isoform of UCA1 is responsible for the sensitization of OAW42‐R cells to cisplatin.

### 3.5 UCA1 is a ceRNA to miR‐27a‐5p, which downregulates UBE2N protein levels to increase BIM expression and sensitize OAW42‐R cells to cisplatin

**Fig. 5:**
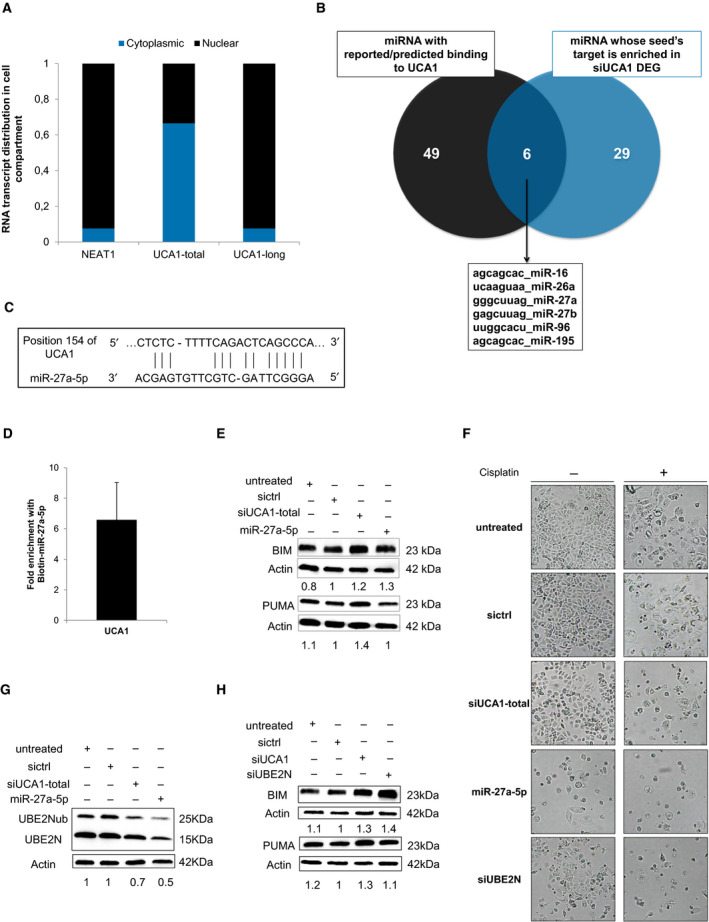
UCA1 inhibition releases miR‐27a‐5p which downregulates UBE2N and induces BIM expression. (A) Relative transcript distribution of UCA1 short and long isoforms (RT‐qPCR) in nuclear and cytoplasmic compartments in OAW42‐R cells normalized to total cellular transcript levels. NEAT1 was used as a control for nuclear transcript. (B) Venn diagram of overlapping miRNAs between miRNAs reported/predicted to bind UCA1 (left) and miRNAs whose seed is enriched in DEGs (right) after UCA1 downregulation in OAW42‐R cells. (C) Schematic representation of short isoform of UCA1 and miR‐27a‐5p sequence complementarity as predicted by RNA22 v2 software. (D) Fold enrichment of UCA1 after Biotin‐miR‐27a‐5p pull‐down relative to Biotin‐Cel‐miR‐67 pull‐down. Data are expressed as mean ± SEM from 3 experiments. (E, G and H) BIM, PUMA, or UBE2N (unmodified and ubiquitinated) protein levels (western blot), quantified relative to actin loading control, representative or mean ± SEM of 3 experiments, after indicated siRNAs transfection in OAW42‐R cells. (F) Pictures from cell layer. Data are representative of 3 experiments

We checked UCA1 subcellular localization by nuclear/cytoplasmic cell fractionation followed by RT‐qPCR, using nuclear localized NEAT1 as a control. While UCA1 long isoform was mainly nuclear, UCA1 short isoform appeared to be cytoplasmic, enabling it to act as a competing endogenous RNA (ceRNA) (Fig. 5A). We then compared (a) all miRNAs reported in the literature or predicted by various algorithms (see Methods section) as being potentially able to bind UCA1 (Fig. S3A), with (b) miRNAs whose seed regions are enriched in the 3’UTR of differentially expressed genes after UCA1 inhibition, as identified by GSEA (Fig. S3A and Appendix S2). The overlap of those 2 lists of miRNAs identified miR‐16, miR‐26a, miR‐27b, miR‐96, miR‐195 and miR‐27a (Fig. 5B and Fig. S3A). Among these, miR‐27a‐5p has sequence complementarity with UCA1 on 13 bases, including a 5 bases stretch from base 2 to base 6 in miR‐27a‐5p seed region, followed by bases 8 and 9 (Fig. 5C). We next investigated whether a direct interaction exists between miR‐27a‐5p and UCA1. We purified RNAs in direct interaction with biotinylated miR‐27a‐5p in OAW42‐R cells. RT‐qPCR revealed a 6‐fold enrichment of UCA1 short isoform after purification on biotinylated miR‐27a‐5p versus biotinylated control, demonstrating direct interaction with miR‐27a‐5p (Fig. 5D).

We next transfected miR‐27a‐5p in OAW42‐R cells, which decreased cell layer confluency to 52% versus 83% in control condition after 48 h (Fig. S3B,C). BIM protein levels were upregulated to an extent similar to what is observed upon siUCA1 transfection, but not PUMA protein levels (Fig. 5E). Six days after transfection, the cell layer in miR‐27a‐5p‐treated cells was greatly reduced (26%) compared to control (96%) and siUCA1 conditions (82%), and sensitization to cisplatin occurred with almost no remaining cells in the culture flask, with 10% confluency compared to 73% and 19% in control and siUCA1 conditions (Fig. 5F and Fig. S3D). This suggests a shared implication of BIM upregulation in sensitizing OAW42‐R cells to cisplatin.

In the set of DEGs after UCA1 inhibition holding 3’UTR sequences complementary to miR‐27a‐5p seed region (Fig. S3E), we looked for potential candidates whose downregulation upon miR‐27a‐5p release from UCA1 might explain the sensitization to cisplatin through modulating BIM expression. Among these is UBE2N whose transcripts were downregulated by 6‐fold (*P* < 0.007) (Appendix S1). UBE2N is a E2 ubiquitin ligase with widespread functions, including in DNA repair by homologous recombination (HR). Interestingly, it had already been demonstrated that UBE2N is a direct target of miR‐27a‐5p [27], which was supported by the enrichment of UBE2N RNA in the pulled down fraction with biotinylated miR‐27a‐5p (Fig. S3F). We then showed that upon miR‐27a‐5p transfection or UCA1 inhibition, total UBE2N protein levels were downregulated (Fig. 5G). We next aimed to determine whether UBE2N inhibition (Fig. S3G) could sensitize OAW42‐R cells to cisplatin and increase BIM expression in the same way as miR‐27a‐5p and UCA1 inhibition. Two days after siUBE2N transfection, the cell layer was moderately reduced compared to the control or siUCA1 conditions, with 64% confluency versus 83% and 75%, respectively (Fig. S3B,C), while 6 days after transfection, the cell layer confluency appeared moderately decreased compared to control and siUCA1 conditions, with 52% confluency versus 96% and 82%, respectively (Fig. 5F and Fig. S3D). However, when siUBE2N transfection was combined with cisplatin, sensitization left almost no remaining cells in the flasks with 16% confluency compared to 73% in control conditions, in a range comparable with siUCA1 (19% confluency) (Fig. 5F and Fig. S3D), which was in line with the observed induction of BIM which occurred 2 days after siUBE2N transfection (Fig. 5H). This suggests that UBE2N inhibition constitutes a relevant strategy to sensitize ovarian cancer cells to cisplatin.

### 3.6 UBE2N inhibitor sensitized patient‐derived organoids to carboplatin

**Fig. 6:**
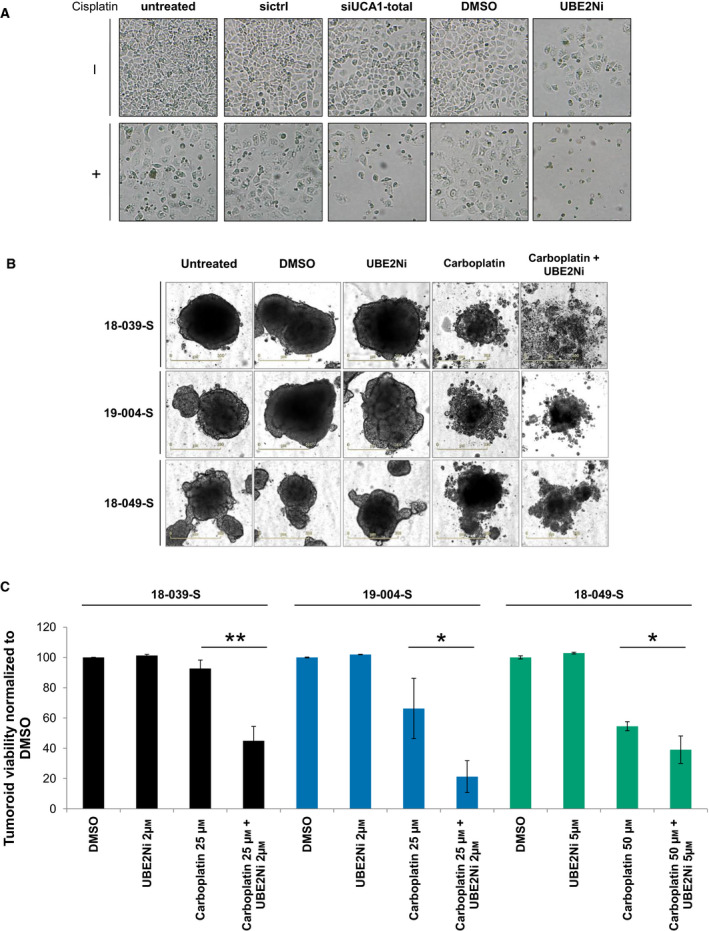
UBE2N inhibitor sensitizes ovarian cancer cell line and patient‐derived organoid lines to platinum salts. (A) Pictures from OAW42‐R cell layer 72 h after indicated siRNA transfection or treatment. Data are representative of 3 experiments. (B, C) Representative pictures and viability of 3 patient‐derived organoid lines after indicated treatments. Viability is normalized to DMSO‐treated control. Data are expressed as mean ± SD of triplicate wells. *P < 0.05; **P < 0.01

We developed several tumor organoid cultures from ovarian cancer patients who displayed an ‘incomplete response’ to platinum‐based therapy according to RECIST criteria, as opposed to ‘complete response’ or ‘progression’. This model is a better proxy of tumor heterogeneity than common cell lines and might faithfully match the response to treatment of the patient of origin [28]. We firstly used a previously validated UBE2N inhibitor, termed UBE2Ni [29] which efficiently sensitized OAW42‐R cells to cisplatin in a range comparable to siUCA1, with 9% and 19% cell confluency, compared to 73% in control condition (Fig. 6A and Fig. S4A). We then assessed the ability of UBE2Ni to sensitize three organoid lines named ’18‐039‐S’, ’19‐004‐S’, and ’18‐049‐S’ to the action of carboplatin, an analogue of cisplatin commonly used in patients. The organoid lines were treated with either 2 or 5 µm of UBE2Ni in combination with increasing doses of carboplatin and cell viability was assessed at the end of the experiment. Treatment with 2 µm UBE2Ni strongly sensitized 18‐039‐S and 19‐004‐S lines to 25 µm carboplatin, with 93% versus 45% cell viability and 66% versus 21% cell viability, respectively (Fig. 6B,C and Fig. S4B–D). However, 5 µm of UBE2Ni only moderately sensitized 18‐049‐S to 50 µm carboplatin with 55% versus 39% cell viability (Fig. 6B,C and Fig. S4E). Altogether these data suggest that UBE2N inhibition could constitute an interesting therapeutic strategy to sensitize ovarian cancer patients to platinum‐based chemotherapy.

## 4 Discussion

**Fig. 7:**
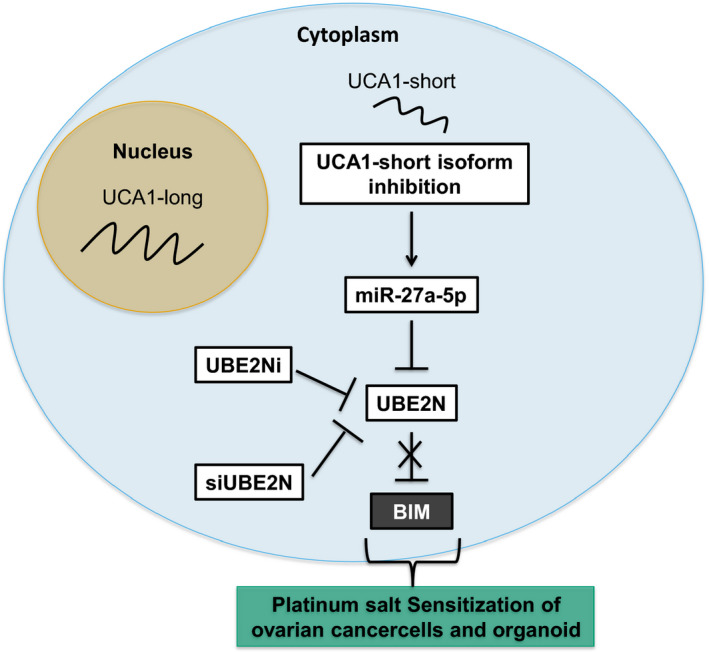
Main findings. UCA1 cytoplasmic short isoform is acting as a competing endogenous RNA for miR‐27a‐5p. Upon UCA1 downregulation, miR‐27a‐5p is no longer prevented to bind and downregulate its direct target UBE2N. UBE2N inhibition triggers BIM upregulation, which sensitizes cells and patient‐derived organoid to the cytotoxic action of platinum drugs

Although an increasing number of studies discuss UCA1 functions in many malignancies [30], the underlying mechanisms are often poorly understood. In this study, we have unraveled a new pathway through which UCA1 exerts oncogenic functions in ovarian cancer (Fig. 7).

There are numerous examples of lncRNAs acting through several different mechanisms, one of the best known is H19, acting both as an epigenetic regulator and as a precursor for miR‐675 [31]. UCA1 has been shown to bind to EZH2 [32], a member of the PRC2 epigenetic regulator complex, or to BRG1 [18], a member of the SWI/SNF chromatin remodeling complex. But UCA1 has been most frequently described to act as a ceRNA, with many miRNAs involved [33, 34, 35].

Several lncRNAs have been reported to display several isoform, up to 38 as has been reported for GNG12‐AS1 [36], and it has been proposed that alternative splicing of lncRNAs can alter their function [37].

It is thus likely that UCA1 different isoforms are at least in part responsible for its varied mechanisms of action. We have shown for the first time that the long isoform (also known as CUDR) is nuclear, while the short isoform is exported to the cytoplasmic compartment, and that they have different functions. Therefore, we can hypothesize that the role of the long isoform would be through its binding of nuclear factors such as EZH2, although its inhibition did not display any phenotype in our study, and that the short isoform would act mainly through its ceRNA functions.

Several studies refer to CUDR specifically, but they mostly perform CUDR inhibition with RNA interference using siRNA or shRNA targeting both isoforms and therefore do not discriminate the functions between them [38, 39].

There are two predicted binding sites for miR‐27a‐5p in UCA1 transcript: one in the common part of both isoforms of UCA1 and another present in the long isoform only. Although we did not formally identify through which of these sites miR‐27a‐5p binds to UCA1, since the long isoform is nuclear, we hypothesize that miR‐27a‐5p binds to UCA1 at the one site present in the short isoform. Interestingly, inhibition of UCA1 long isoform by specific siRNAs also decreased the levels of UCA1 short isoform by around 50%, while not sensitizing cells to cisplatin. UCA1 in OAW42‐R cells is highly expressed, whereas miRNAs are in general expressed at low or moderate copy number in cells. Because UCA1 and miR‐27a‐5p should interact through a 1 : 1 a stochiometric relation, we hypothesize that around 50% of UCA1 copies is still enough to buffer endogenous miR‐27a‐5p, whereas its quite strong downregulation by 95% with specific siRNAs will prevent sequestration of enough miR‐27a‐5p to impair its action on targeted mRNAs.

According to the set of differentially expressed genes after UCA1 downregulation, several miRNAs might be bound to UCA1 and released upon its downregulation, destabilizing targeted mRNA transcripts. We however showed that miR‐27a‐5p transfection mimics UCA1‐induced BIM induction, which we have shown to be a necessary condition for ovarian cancer cell sensitization to cisplatin. Its recovered ability to target mRNAs after UCA1 upon downregulation (miR‐27a‐5p is expressed in the ovarian cancer cells we used in this study—data not shown) is therefore the main event leading to the upregulation of BIM, a proapoptotic member of the BCL2 family whose upregulation sensitizes cells to various chemotherapeutic agents [40]. Upregulation of PUMA, which like BIM can sequester most of antiapoptotic members of the BCL2 family, does not sensitize to cisplatin in our model. This suggests that it is the superior ability of BIM, compared to PUMA [41], to activate multidomain proapoptotic members, BAX and/or BAK which is responsible for the observed sensitization.

It has been shown that miR‐27a‐5p has potential tumor suppressive function in lung adenocarcinoma [42] and small cell lung cancer [43]. It also targets EGFR in head and neck cancer [44], which is moderately downregulated in our model after siUCA1 transfection (1,42 fold). Interestingly, we have shown previously that EGFR inhibition upregulates BIM in ovarian cancer cells [45]. We however demonstrated that UBE2N, which has been shown previously to be a direct target of miR‐27a‐5p [27], is downregulated by UCA1 inhibition or miR‐27a‐5p transfection, which triggers BIM upregulation to an extent comparable to UCA1 inhibition. EGFR moderate inhibition in response to UCA1 downregulation might add to UBE2N inhibition for inducing BIM expression, but does not constitute the main contribution.

There are two possible pathways through which the E2 ubiquitin ligase UBE2N downregulation might lead to BIM upregulation. First, UBE2N in the cytoplasm is bound to Uev1A and can ubiquitinylate IRAK1 and TRAF6, leading to IKK phosphorylation and subsequent NF‐kB activation [46]. UBE2N inhibition might then promote BIM upregulation through NF‐kB inhibition [47]. Second, UBE2N in the nucleus is an important mediator of the DNA damage response to double‐strand breaks by HR [48]. UBE2N inhibition leads to genomic instability and increases the persistence of DNA damage, which is known to trigger BIM upregulation [49].

UBE2N has been proposed as a promising target in melanoma xenografts [50] and large B‐cell lymphoma cells [29]. It has also been suggested recently in a study in ovarian cancer cells that a decrease in UBE2N expression could promote resistance to Paclitaxel [51]. However, in the platinum/taxane association constituting the standard chemotherapy in ovarian cancer patients, it is the platinum‐free interval which is often used to assess patient’s prognosis [52]. Interestingly, we have shown that UBE2N inhibition efficiently sensitizes to platinum salt patient‐derived organoid of ovarian cancer. These models are more faithful than typical monolayer cell lines to mimic the response to treatment for a number of malignancies, including ovarian cancer [53], and they constitute highly promising tools to predict patients’ response to treatment. Moreover, patients from whom we derived the organoid cultures used in this study did show an incomplete response to platinum‐based chemotherapy, suggesting that UBE2N inhibition might constitute a promising strategy to overcome resistance to current treatments, which is a major cause of therapeutic failure in ovarian cancer.

In addition, NF‐kB activation upregulates PDL1, a negative regulator of immune response to tumor cells [54], and UBE2N inhibition might then help elicit immune‐mediated antitumor response *in vivo*, or sensitize to anti‐PDL1 therapies currently studied in ovarian cancer [55]. Moreover, UBE2N inhibition and subsequent impairment of HR DNA repair pathway could sensitize to the newly available PARPi molecules [56], whose activity relies on defects in HR.

## 5 Conclusions

To conclude, this study unravels the function and mode of action of UCA1 in the response to treatment in ovarian cancer cells and therefore emphasizes on the pivotal roles of lncRNAs in mechanisms of response to treatment in cancer. More importantly, our work in patient‐derived organoids enlightens UBE2N as a highly promising target, paving the way to a new potential therapeutic strategy for the treatment of ovarian cancer.

## Conflict of interest

The authors declare no conflict of interest.

## Author contributions

Conceptualization, CD and MMF; Methodology, MMF, PMM and LBW; Investigation, AW, MA, PMM, EA, EB, MF and MMF; Resources, LBW; Writing—Original Draft, AW and MMF; Writing—Review and Editing, AW, MA, PMM, BL, LBW, MV, NV, PG, LP, CD and MMF; Supervision, CD and MMF; Funding Acquisition, MMF.

### Peer Review

The peer review history for this article is available at https://publons.com/publon/10.1002/1878‐0261.13045.

## Supporting information


**Fig. S1**. UCA1 inhibition sensitizes to cisplatin OAW42 and OVCAR3 cell lines.
**Fig. S2**. Cisplatin sensitization through UCA1 downregulation relies on Bim upregulatio.
**Fig. S3**. UCA1 inhibition releases miR‐27a‐5p which downregulated UBE2N and induces BIM.
**Fig. S4**. UBE2N inhibition sensitizes OAW42‐R cells and patient‐derived organoid lines to platinum salts.
**Table S1**. siRNAs and miRNA sequences.
**Table S2**. Primer sequences.
**Appendix S1**. Microarray data siUCA1 versus sictrl. Differential expression fold changes, p‐values and q‐values for all detected transcripts.
**Appendix S2**. miRNA seed sequences enriched in DEGs after siUCA1 transfection. Recapitulates all the miRNAs whose seed sequences were enriched in the 3’UTR of genes differentially expressed after UCA1 downregulation.
**Appendix S3**. Assembles all supplementary figures and tables and associated legends, including.

## Data Availability

Source data of microarray for the experiment exploring the effects of UCA1 downregulation have been deposited on GEO and are accessible in the following repository: GSE153087. Source data of microarray comparing basal transcriptomes between OAW42 and OAW42‐R cell lines have been deposited on GEO and are accessible in the following repository: GSE162538.
