# Supplementary material for: The lncRNA ‘UCA1’ modulates the response to chemotherapy of ovarian cancer through direct binding to miR‐27a‐5p and control of UBE2N levels
Source: Mol Oncol. 2021 Jul 13;15(12):3659–78. doi: 10.1002/1878-0261.13045 (PMC8637575; doi:10.1002/1878-0261.13045)
Supplement: Supplementary file 1 — Fig. S1. UCA1 inhibition sensitizes to cisplatin OAW42 and OVCAR3 cell lines. Fig. S2. Cisplatin sensitization through UCA1 downregulation relies on Bim upregulatio. Fig. S3. UCA1 inhibition releases miR‐27a‐5p which downregulated UBE2N and induces BIM. Fig. S4. UBE2N inhibition sensitizes OAW42‐R cells and patient‐derived organoid lines to platinum salts. Table S1. siRNAs and miRNA sequences. Table S2. Primer sequences. Appendix S1. Microarray data siUCA1 versus sictrl. Differential expression fold changes, p‐values and q‐values for all detected transcripts. Appendix S2. miRNA seed sequences enriched in DEGs after siUCA1 transfection. Recapitulates all the miRNAs whose seed sequences were enriched in the 3’UTR of genes differentially expressed after UCA1 downregulation. Appendix S3. Assembles all supplementary figures and tables and associated legends, including. [file MOL2-15-3659-s001.pdf]

Supplementary Table 1. siRNAs and miRNA sequences

Target	siRNA	Target sequence (5'-3')
**Short/long isoform UCA1**	siUCA1(2)	5'-cacccuagcuggacgauca-3'
**Short/long isoform UCA1**	siUCA1(4)	5'-gauuaggccgagagccgau-3'
**Long isoform of UCA1**	siUCA(3)	5'-acccuagacccgaaacuua-3'
**UBE2N**	siUBE2N	5'-gcacaguucugcuaucgau-3'
**BIM**	siBIM	5'- gguuaucuuacgacuguua-3'
**PUMA**	siPUMA	5'-uauacaguaucuuacaggctt-3'
**Cel-miR-67-mimic**	Cel-miR-67	5'-ucacaaccuccuagaaagaguaga-3"
**hsa-miR-27a-5p-mimic**	miR-27a-5p	5'-agggcuuagcugcuugugagca-3'

Supplementary Table 2, primer sequences

Target	Primers	Sequence (5’-3’)
UCA1c short/long isoform	Forward	5’-cagatccttgcccatggtgt-3’
	Reverse	5’-ctgaggctggcaaagagtga-3’
UCA1lg long isoform	Forward	5’-gacaggaacctcaacccaaa-3’
	Reverse	5’-aaaccacatgaaacgcctct-3’
SDHA	Forward	5’-tgggaacaagagggcatctg-3’
	Reverse	5’-ccaccactgcatcaaattcatg-3’
BIM	Forward	5’-gctttgccgcccttatgatg-3’
	Reverse	5’-gcctgccagactgagtttct-3’
PUMA	Forward	5’-ccaaacgtgaccactagcct-3’
	Reverse	5’-gatgaaggtgaggcaggcat-3’
UBE2N	Forward	5’-aacgagtgccagtgtgttga-3’
	Reverse	5’-ttttcaactgctgccttggc-3’

Supplementary File 1. Microarray data siUCA1 versus sictrl

See corresponding file

Supplementary File 2. miRNA seed sequences enriched in DEGs after siUCA1 transfection.

See corresponding file

Supplementary Figure 1. UCA1 inhibition sensitizes to cisplatin OAW42 and OVCAR3 cell lines

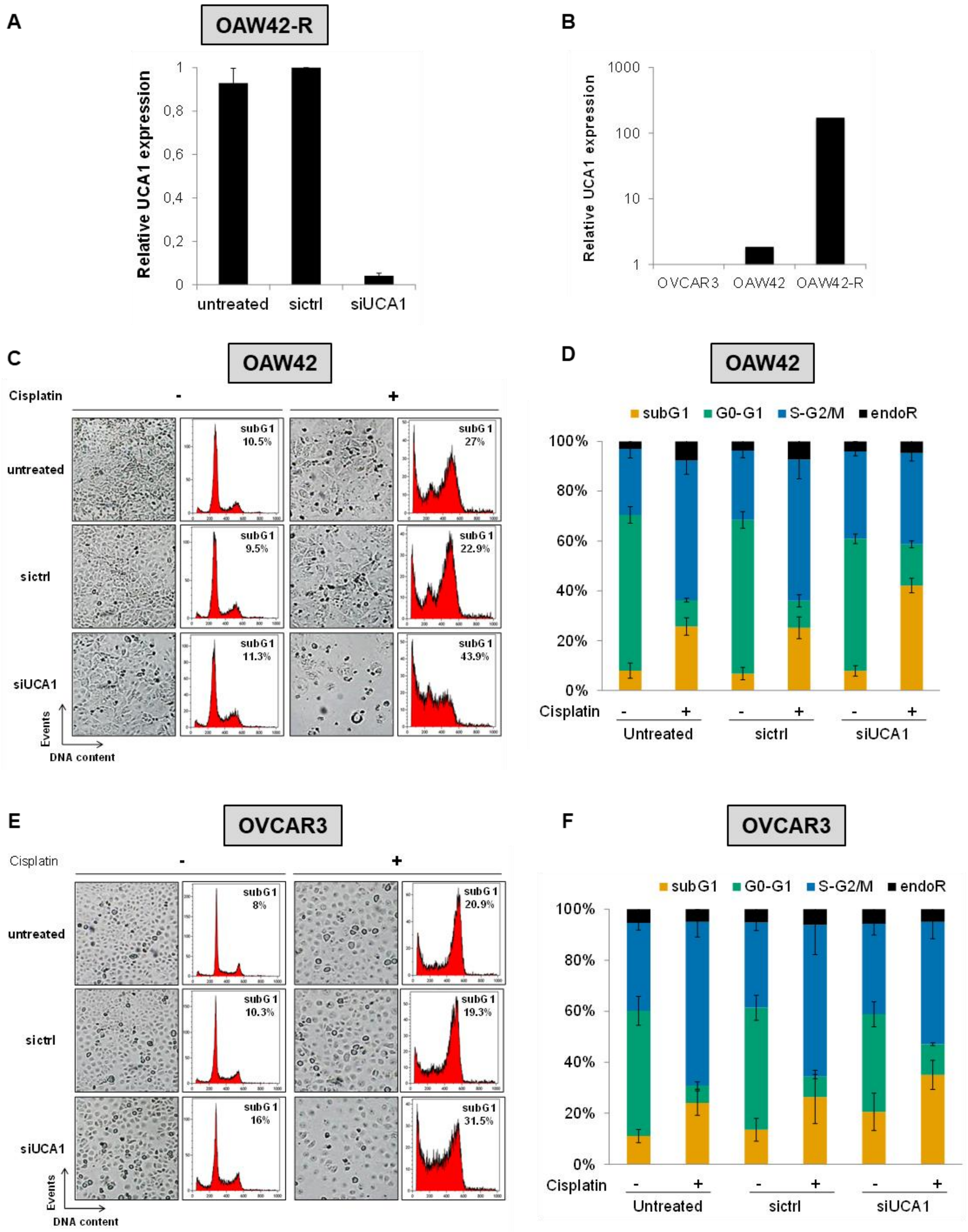

Supplementary Figure 1. UCA1 inhibition sensitizes to cisplatin OAW42 and OVCAR3 cell lines

(**A**) UCA1 transcript inhibition (RT-qPCR) after transfection with siUCA1. (**B**) UCA1 expression levels (RT-qPCR) in OVCAR3, OAW42 and OAW42-R cell lines, relative to OVCAR3, normalized with SDHA. (**C**) OAW42 cells: picture from cell layer and DNA content profiles. Representative pictures and profiles from 3 experiments are shown. (**D**) OAW42 cells: DNA content histograms expressed as mean ± SEM of at least three independent experiments. (**E**) OVCAR3 cells: picture from cell layer and DNA content profiles. Representative pictures and profiles from 3 experiments are shown. (**F**) OVCAR3 cells: DNA content histograms expressed as mean ± SEM of at least three independent experiments.

Supplementary Figure 2. Cisplatin sensitization through UCA1 downregulation relies on Bim up-regulation

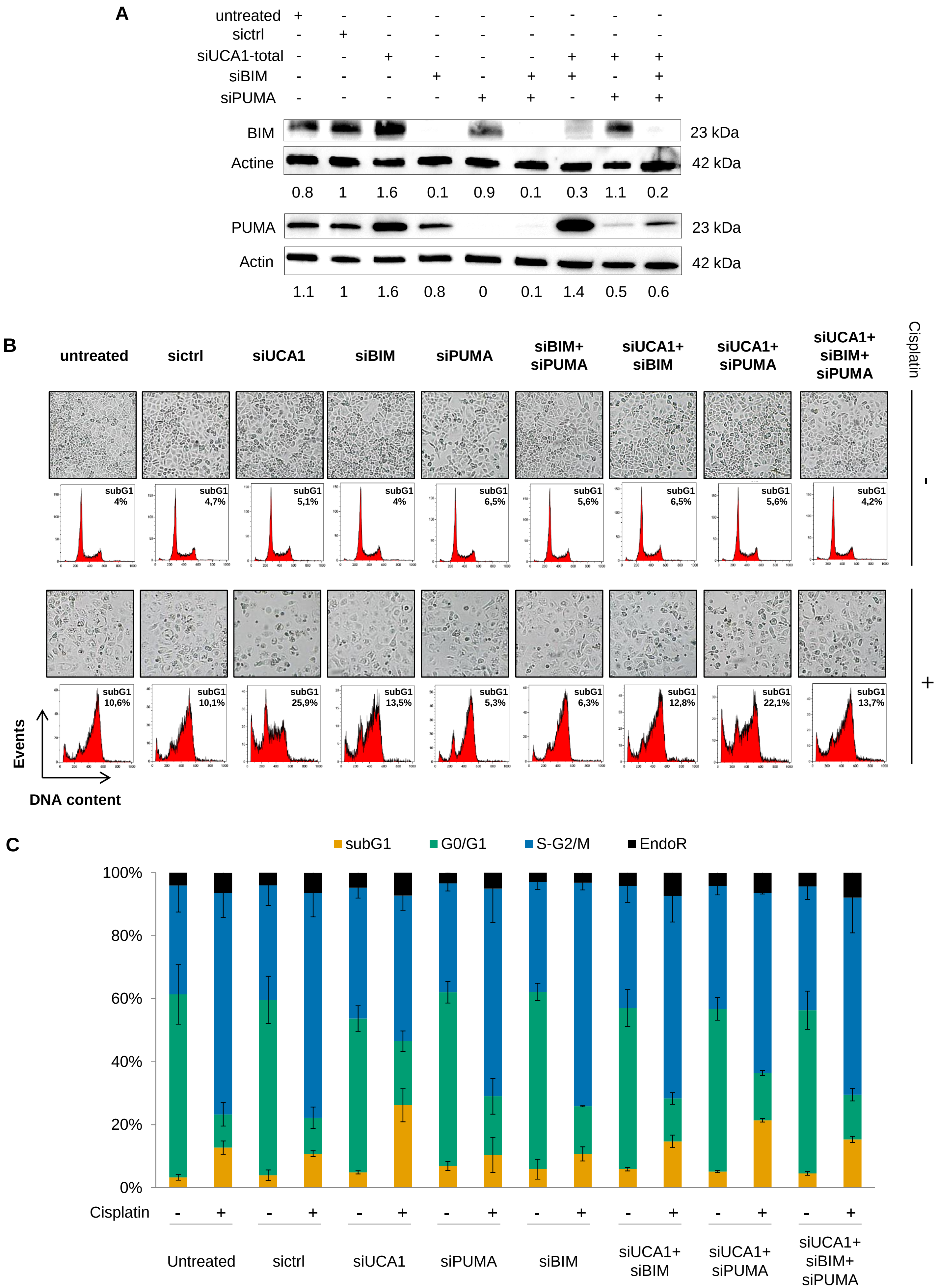

Supplementary Figure 2. Cisplatin sensitization through UCA1 downregulation relies on BIM up-regulation.

(**A**) BIM and PUMA protein levels (western blot), quantified relative to actin loading control, representative or mean +/- SEM of 3 experiments, after indicated siRNAs transfection in OAW42-R cells. (**B**) Picture from cell layer and DNA content profiles. Representative pictures and profiles are shown. (**C**) DNA content histograms expressed as mean ± SEM of 3 experiments.

Supplementary Figure 3. UCA1 inhibition releases miR-27a-5p which downregulates UBE2N and induces BIM

A

miRNA with reported/predicted binding to UCA1					miRNA whose seed's target is enriched in siUCA1 DEG				overlap
miR-1	miR-135a	miR-214	miR-582	miR-4640	miR-15a	**miR-27b**	miR-181a	miR-519c	**miR-16**
**miR-16**	miR-143	miR-216b	miR-613	miR-4644	miR-15b	miR-30a	miR-181b	miR-519d	**miR-26a**
miR-18a	miR-145	miR-296	miR-624	miR-4726	**miR-16**	miR-30b	miR-181c		**miR-27a**
miR-18b	miR-182	miR-331	miR-665	miR-4735	miR-17	miR-30c	miR-181d		**miR-27b**
**miR-26a**	miR-184	miR-429	miR-873	miR-4761	miR-19a	miR-30d	**miR-195**		**miR-96**
**miR-27a**	miR-185	miR-485	miR-1271	miR-4770	miR-19b	miR-30e	miR-301		**miR-195**
**miR-27b**	miR-193a	miR-495	miR-1914	miR-5000	miR-20a	**miR-96**	miR-424		
**miR-96**	**miR-195**	miR-499b	miR-2467	miR-5194	miR-20b	miR-106a	miR-497		
miR-99b	miR-203	miR-507	miR-4306	miR-6088	**miR-26a**	miR-106b	miR-506		
miR-122	miR-204	miR-513a	miR-4436a	miR-6738	miR-26b	miR-130a	miR-519a		
miR-129	miR-206	miR-513b	miR-4510		**miR-27a**	miR-130b	miR-519b		

B

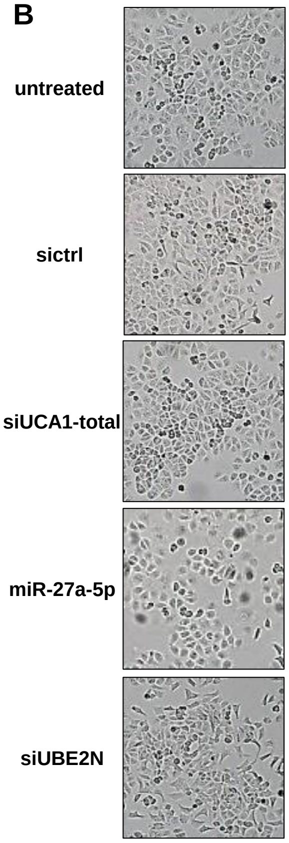

E

DEGs after UCA1 downregulation bearing miR-27a-5p seeds in their 3'UTR region		
SPTY2D1	AK2	CSRNP1
PARD6B	KCNK2	NAV2
CAMTA1	AMMECR1L	C11orf57
ABCA1	FLRT3	AGPAT4
ZNF800	CTDSP2	RMND5A
TMCC1	LIF	RGPD5
FAM134C	CBFA2T3	GCC2
SIPA1L3	ATP11C	GOSR2
DNAJC27	DNAJC13	CD2AP
UNKL	CCNK	CCNG1
N4BP1	TSC1	MAP2K7
E2F7	ARMC8	**UBE2N**
PGM2L1	CLCN5	C17orf85
MYT1	PPARG	DOT1L
ENAH	ZHX1	RNFT1
BTG2	RELN	PPME1
ZCCHC24	GATAD2A	ELFN2
SH3RF1	NEK6	ING5
SLC7A11	HBEGF	**UBE2NL**
APPBP2	SFRP1	PANK1
B4GALT3	EGFR	SLC39A11
AQP11	INSR	

F

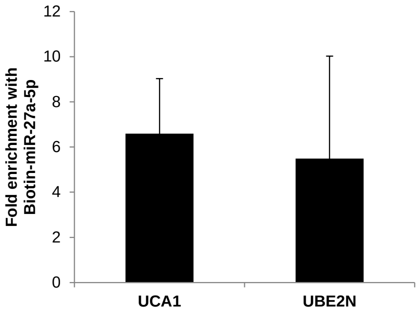

G

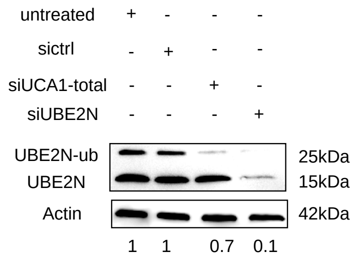

C

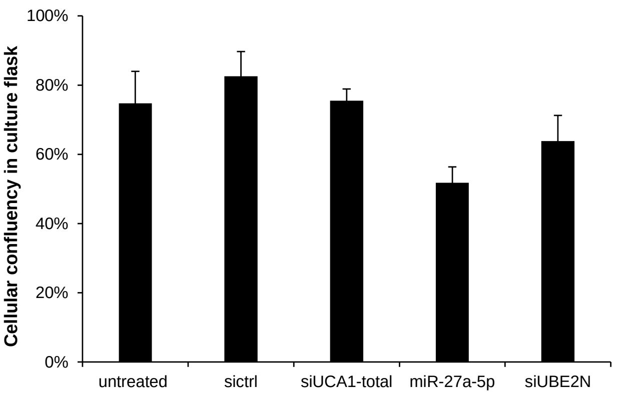

D

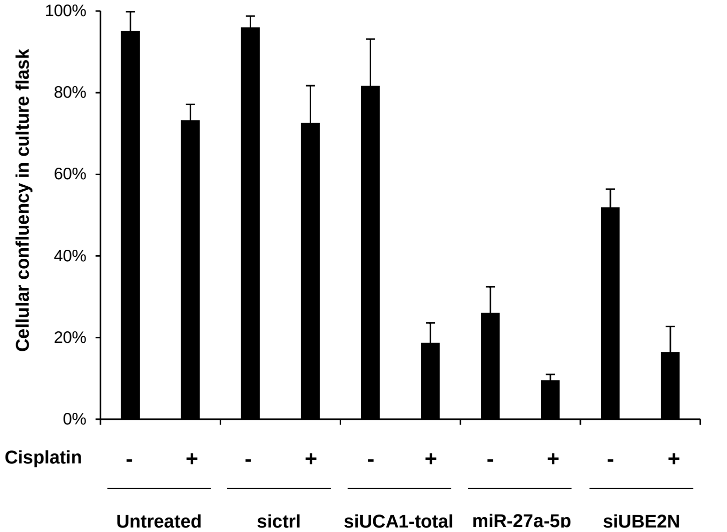

Supplementary Figure 3. UCA1 inhibition releases miR-27a-5p which downregulates UBE2N and induces BIM

(**A**) Table showing the full list of miRNAs from the Venn diagram in Figure 5B. (**B**) Pictures from cell layer 48 h after transfection with indicated siRNAs or miRNA. Data are representative of 3 experiments. (**C**) Table showing DEGs after UCA1 downregulation bearing miR-27a-5p seed complementarity in their 3'UTR. (**D**) Fold enrichment of UCA1 and UBE2N after Biotin miR-27a-5p pull-down relative to Biotin Cel-miR-67 pull-down. Data are expressed as mean +/- SEM from 3 experiments (**E**) UBE2N (unmodified and ubiquitinylated) protein levels (western blot), quantified (lower lines) with actin, representative or mean +/- SEM of 3 experiments, after indicated siRNAs transfection in OAW42-R cells.

Supplementary Figure 4. UBE2N inhibition sensitizes OAW42-R cells and patient-derived organoid lines to platinum salts

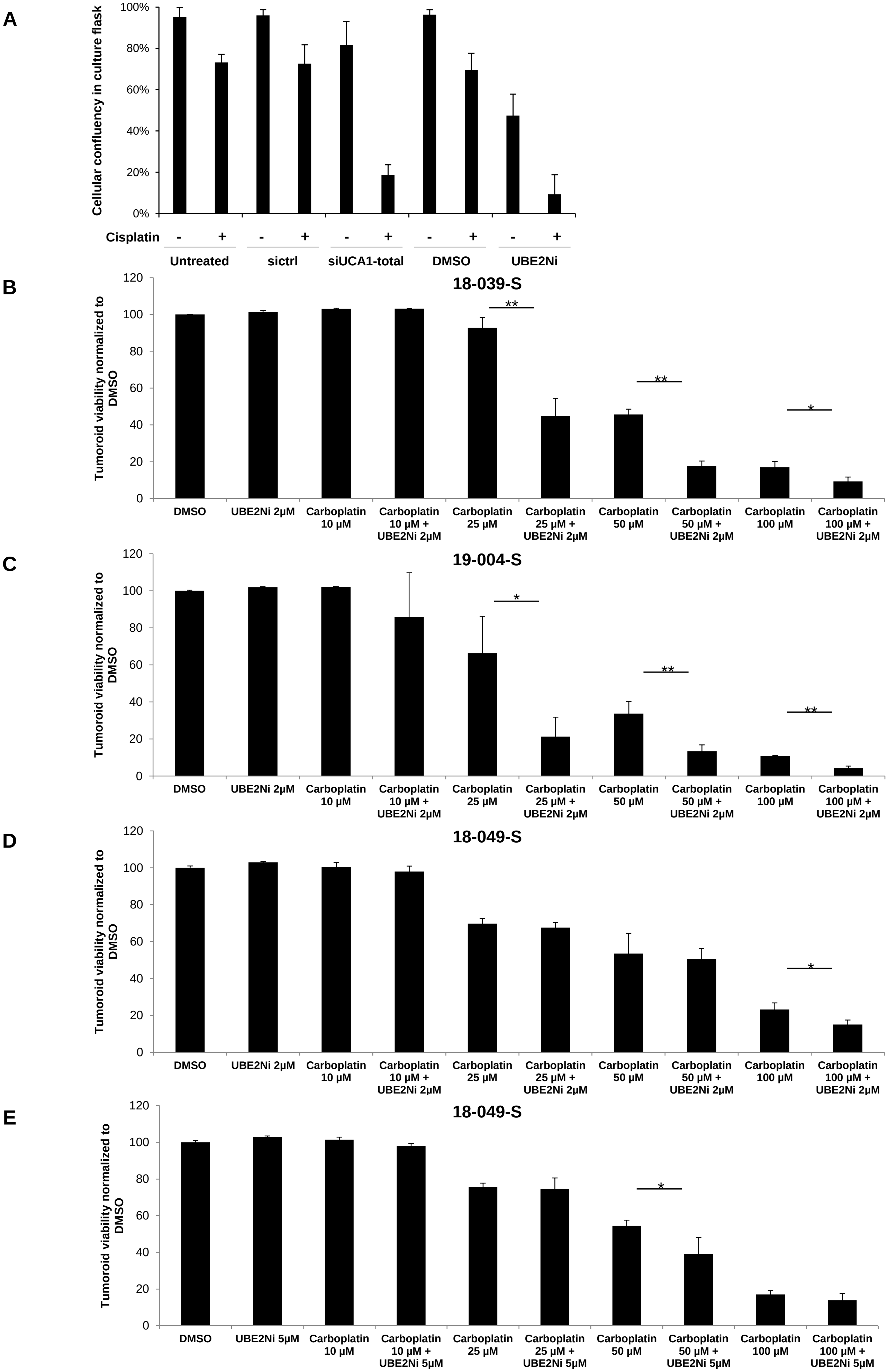

Supplementary Figure 4. UBE2N inhibition sensitizes OAW42-R cells and patient-derived organoid lines to platinum salts

Measurement of cell confluency in OAW42-R cells after indicated treatments. Data are expressed as mean +/- SD of 3 experiments (**A**).Viability of 3 patient-derived organoid lines after escalading concentrations of carboplatin. (**B**, **C** and **D**) UBE2N inhibitor concentration is 2 µM, (**E**) UBE2N concentration is 5 µM. Viability is normalized to DMSO treated control. Data are expressed as mean +/- SD of triplicate wells. $p < 0.05$ was noted as "*", and $p < 0.01$ was noted as "**".
